# Quantifying the breadth of antibiotic exposure in sepsis and suspected infection using spectrum scores

**DOI:** 10.1097/MD.0000000000030245

**Published:** 2022-10-14

**Authors:** Joshua T. Smith, Raj N. Manickam, Fernando Barreda, John D. Greene, Meghana Bhimarao, Jason Pogue, Makoto Jones, Laura Myers, Hallie C. Prescott, Vincent X. Liu

**Affiliations:** a Pharmacy Quality and Medication Safety, Kaiser Permanente Northern California, Oakland, CA; b Division of Research, Kaiser Permanente Northern California, Oakland, CA; c College of Pharmacy, University of Michigan, Ann Arbor, MI; d Division of Epidemiology, VA Salt Lake City Health Care System, Salt Lake City, UT; e Division of Epidemiology, University of Utah, Salt Lake City, UT; f Department of Internal Medicine, University of Michigan, Ann Arbor, MI; g VA Center for Clinical Management Research, Ann Arbor, MI.

**Keywords:** antibiotics, antimicrobial stewardship, drug combinations, health care, mortality, quality indicators, sepsis

## Abstract

A retrospective cohort study. Studies to quantify the breadth of antibiotic exposure across populations remain limited. Therefore, we applied a validated method to describe the breadth of antimicrobial coverage in a multicenter cohort of patients with suspected infection and sepsis. We conducted a retrospective cohort study across 21 hospitals within an integrated healthcare delivery system of patients admitted to the hospital through the ED with suspected infection or sepsis and receiving antibiotics during hospitalization from January 1, 2012, to December 31, 2017. We quantified the breadth of antimicrobial coverage using the Spectrum Score, a numerical score from 0 to 64, in patients with suspected infection and sepsis using electronic health record data. Of 364,506 hospital admissions through the emergency department, we identified 159,004 (43.6%) with suspected infection and 205,502 (56.4%) with sepsis. Inpatient mortality was higher among those with sepsis compared to those with suspected infection (8.4% vs 1.2%; *P* < .001). Patients with sepsis had higher median global Spectrum Scores (43.8 [interquartile range IQR 32.0–49.5] vs 43.5 [IQR 26.8–47.2]; *P* < .001) and additive Spectrum Scores (114.0 [IQR 57.0–204.5] vs 87.5 [IQR 45.0–144.8]; *P* < .001) compared to those with suspected infection. Increased Spectrum Scores were associated with inpatient mortality, even after covariate adjustments (adjusted odds ratio per 10-point increase in Spectrum Score 1.31; 95%CI 1.29–1.33). Spectrum Scores quantify the variability in antibiotic breadth among individual patients, between suspected infection and sepsis populations, over the course of hospitalization, and across infection sources. They may play a key role in quantifying the variation in antibiotic prescribing in patients with suspected infection and sepsis.

## 1. Introduction

Sepsis has a staggering impact worldwide, contributing to up to 20% of all global deaths.^[1]^ In the US, sepsis is the single most common cause of hospital death playing a role in the deaths of up to 16% of patients annually.^[2–4]^ The early identification of at-risk patients and initiation of appropriate antibiotics remain the cornerstone of treatment for patients with suspected sepsis.^[5,6]^ As a result, the past decade has seen significant growth in performance improvement, quality reporting, and public education campaigns focused on accelerating antibiotic administration in sepsis.^[7]^

However, numerous concerns have been raised about the intense focus on the use of empiric, broad-spectrum, antibiotics. Indiscriminate antimicrobial use can lead to antimicrobial resistance as well as adverse sequelae for individual patients including *Clostridioides difficile* (*C. difficile*) infection or kidney injury.^[8–11]^ In both the inpatient and outpatient settings, recent studies highlight significant concerns about overuse, suggesting that current practices might be increasing the cumulative population-level exposure to inappropriate antibiotics.^[12–15]^ Despite these concerns, metrics to quantify the breadth of antibiotic exposure across populations remain limited, with variable definitions of broad-spectrum antibiotic regimens across studies.^[15–17]^

In this study, we applied a previously developed metric for quantifying antibiotic breadth, the Spectrum Score, to a contemporary, multicenter population of inpatients treated for infection and sepsis. We hypothesized that the Spectrum Score could be used to demonstrate variability in antibiotic prescribing across potentially infected inpatients, facilitating a quantitative approach to measuring, comparing, and evaluating antibiotic practices in the research setting. The Spectrum Score could also be used as a tool for infectious disease specialists working in antibiotic stewardship to identify patients receiving inappropriately broad-spectrum antibiotics with the goal of limiting patients’ unnecessary exposure to broad-spectrum antibiotics and preventing resistance. As a means to demonstrate face validity of the tool, we also assessed whether the Spectrum Score was associated with inpatient mortality.

## 2. Methods

This retrospective cohort study was approved by the Kaiser Permanente Northern California (KPNC) Institutional Review Board (#1489470-1) with a waiver of informed consent.

### 2.1. Suspected infection and sepsis-3 cohorts

We used data extracted from the electronic health records of nonsurgical, nonobstetric patients admitted to 21 hospitals in the KPNC integrated healthcare delivery system, which serves 4.5 million members. We identified all hospitalizations admitted through the emergency department (ED) among adult patients (aged ≥ 18 years) with suspected infection (SI) or sepsis (S3) based on the Sepsis-3 criteria between 2012 and 2017.^[18–21]^ We identified *suspected infection* patients based on the timed dyad of antibiotics and microbiologic cultures and, thereafter, identified *sepsis* patients using a Sequential/Sepsis-related Organ Failure Assessment (SOFA) score of ≥ 2 based on the Sepsis-3 consensus definitions.^[22]^

We identified all hospitalizations admitted through the emergency department (ED) among adult patients (aged ≥ 18 years) with suspected infection (SI) or sepsis (S3) based on the Sepsis-3 criteria between 2012 and 2017.^[18–21]^ We identified *suspected infection* patients based on the timed dyad of antibiotics and microbiologic cultures and, thereafter, identified *sepsis* patients using a Sequential/Sepsis-related Organ Failure Assessment (SOFA) score of ≥ 2 based on the Sepsis-3 consensus definitions.^[22]^

We identified all hospitalizations admitted through the emergency department (ED) among adult patients (aged ≥ 18 years) with suspected infection (SI) or sepsis (S3) based on the Sepsis-3 criteria between 2012 and 2017.^[18–21]^ We identified *suspected infection* patients based on the timed dyad of antibiotics and microbiologic cultures and, thereafter, identified *sepsis* patients using a Sequential/Sepsis-related Organ Failure Assessment (SOFA) score of ≥ 2 based on the Sepsis-3 consensus definitions.^[22]^

### 2.2. Spectrum score

The Spectrum Score, originally described by Madaras-Kelly and others, was developed in a 3-stage Delphi process to quantify the spectrum of antimicrobial activity for antibiotic regimens using U.S. Veteran’s Affairs susceptibility data.^[23]^ The Spectrum Score is a numeric value, ranging between 0 and 64, with higher scores indicating broader antibiotic coverage against 14 organism domains (e.g., *Staphylococcus aureus*, *Escherichia coli*, and *Pseudomonas aeruginosa*) from 27 antibacterial groups (e.g., 3^rd^ generation cephalosporins, macrolides, antipseudomonal fluoroquinolones, nonpseudomonal fluoroquinolones).^[24]^ Scores are assigned based on susceptibility data quintiles (e.g., 0 points for susceptibilities of < 20% and 4 points for susceptibilities ≥ 80%) with extra weight for intrinsically resistant organisms (e.g., *Pseudomonas aeruginosa* scores are multiplied by 1.75). Double coverage with combination antibiotic therapy was penalized using an assumption of independently distributed co-resistance. The validity of the Spectrum Score values were assessed and confirmed by Delphi participants in a series of clinical vignettes comparing Spectrum Score changes to expert review.

We identified antibiotic administration including medication name, route, and frequency of administration from the medication administration record, including only those given through intravenous and digestive tract routes. Antibiotics that were administered in the ED were included within the first daily score.

Using the Spectrum Score, we calculated the additive and global scores for each hospitalization starting at the time of hospital admission (Table 1). To calculate the global Spectrum Score, we identified the unique combination of antibiotic classes administered throughout each hospital encounter and computed a score (Table 1, Supplemental Digital Content, http://links.lww.com/MD/H100). To calculate the additive Spectrum Score, we divided every hospitalization into 24-hour intervals based on time of hospital admission and summed together each 24-hour score. We grouped hospitalizations into quartiles based on their global Spectrum Scores and defined broad-spectrum antibiotic regimens as those whose global values were ≥ 75^th^ percentile (Table 2, Supplemental Digital Content, http://links.lww.com/MD/H100).

**Table 1:** Characteristics of patients hospitalized with suspected infection and sepsis.

Characteristic	Both	Suspected infection	Sepsis	*P* value
	(n = 364,506)	(n = 159,004)	(n = 205,502)	
Unique patients	218,215	117,751	130,087	
Age, median (IQR), years	71.0 (58.0,82.0)	68.0 (53.0,80.0)	73.0 (62.0,83.0)	<.001
Male, n (%)	170,871 (46.9)	65,067 (40.9)	105,804 (51.5)	<.001
Race, n (%)				<.001
White	212,506 (58.5)	95,825 (60.5)	116,681 (57.0)	
Hispanic	54,539 (15.0)	24,167 (15.3)	30,372 (14.8)	
Asian	36,853 (10.1)	14,408 (9.1)	22,445 (11.0)	
Black	35,605 (9.8)	14,139 (8.9)	21,466 (10.5)	
Other	23,613 (6.5)	9765 (6.2)	13,848 (6.8)	
COPS2, median (IQR)	42.0 (16.0,77.0)	27.0 (10.0,57.0)	55.0 (26.0,90.0)	<.001
LAPS2, median (IQR)	77.0 (52.0,106.0)	60.0 (40.0,83.0)	92.0 (67.0,120.0)	<.001
Length of stay, median (IQR), hours	78.0 (46.7137.4)	66.4 (42.1110.7)	91.3 (56.1160.9)	<.001
Direct ICU admission, n (%)	49,630 (13.6)	7095 (4.5)	42,535 (20.7)	<.001
Inpatient mortality, n (%)	19,173 (5.3)	1953 (1.2)	17,220 (8.4)	<.001
Admission care order, n (%)				<.001
Full code	285,354 (78.3)	131,279 (82.6)	154,075 (75.0)	
DNR	71,610 (19.6)	25,358 (15.9)	46,252 (22.5)	
Partial code	7443 (2.0)	2338 (1.5)	5105 (2.5)	
Comfort care	99 (0.0)	29 (0.0)	70 (0.0)	
Infection source, n (%)				<.001
Mixed	114,786 (44.7)	42,309 (40.3)	72,477 (47.8)	
Respiratory	55,914 (21.8)	24,251 (23.1)	31,663 (20.9)	
Bone, skin, or soft tissue	26,225 (10.2)	14,382 (13.7)	11,843 (7.8)	
Genitourinary	25,764 (10.0)	10,095 (9.6)	15,669 (10.3)	
Other	23,217 (9.0)	9751 (9.3)	13,466 (8.9)	
Gastrointestinal	9613 (3.7)	3656 (3.5)	5957 (3.9)	
Central Nervous System	1210 (0.5)	667 (0.6)	543 (0.4)	
Number of Antibiotic Classes, median (IQR)	2.0 (1.0,3.0)	2.0 (1.0,2.0)	2.0 (1.0,3.0)	<.001
Days on antibiotics, median (IQR)	3.0 (2.0,5.0)	3.0 (2.0,4.0)	3.0 (2.0,5.0)	<.001
Global spectrum score, mean (SD)	40.1 (13.2)	38.4 (13.1)	41.4 (13.1)	<.001
Global spectrum score, median (IQR)	43.8 (29.2,49.0)	43.5 (26.8,47.2)	43.8 (32.0,49.5)	<.001
Additive spectrum score, mean (SD)	146.5 (176.5)	120.6 (136.0)	166.5 (200.00)	<.001
Additive spectrum score, median (IQR)	96.0 (51.0,178.5)	87.5 (45.0,144.8)	114.0 (57.0,204.5)	<.001

COPS2 = Comorbidity Point Score = version 2, DNR = do not resuscitate, ED = emergency department, ICU = intensive care unit, IQR = interquartile range, LAPS2 = Laboratory Acute Physiology Score = version 2.

### 2.3. Patient characteristics

We described patients by baseline hospital characteristics including age, gender, race/ethnicity, COPS2 (a previously validated, scalar *Comorbidity Point Score, version 2* ranging from 0-1014 which evaluates all comorbid diagnoses from the prior year), admission care order status ranging from full code to comfort care, first inpatient unit, and admission category (medical vs surgical).^[25]^ Admission care order status, or code status orders, reflect patient or proxy wishes for life support treatments (e.g., defibrillation, vasopressors, endotracheal intubation, dialysis, transvenous pacing, among others). At KPNC, partial code reflects patient preferences for only specific types of life support therapy. We also assessed acute severity of illness metrics with the LAPS2 (*Laboratory and Acute Physiology score, version 2*), a score ranging from 0 to 414 including vital signs, 16 laboratory results, and neurologic status in the 72 hours preceding hospitalizations.^[25,26]^ Patients with any missing values were minimal (< 0.5%) and removed from analysis.

To evaluate Spectrum Scores across patient subgroups, we grouped patients by potential infection source using anatomical or pathophysiologic categories, including: (1) bone, skin, or soft tissue; (2) central nervous system, (3) gastrointestinal, (4) genitourinary, (5) respiratory, and (6) other using Healthcare Cost and Utilization Project Clinical Classification Software (CCS) single-level groups based on primary and secondary International Classification of Disease diagnosis codes (Table 3, Supplemental Digital Content, http://links.lww.com/MD/H100).^[27,28]^ We classified hospitalizations with only a single infection source diagnosis code in any position within that source, and those with multiple sources of infection as a distinct “mixed” group.

### 2.4. Statistical analysis

Data are presented as number (%), mean ± standard deviation, or median (interquartile range, IQR). We compared baseline characteristics between groups using Student t-tests, Kruskal-Wallis tests, or chi-squared tests. To assess whether patients’ Spectrum Scores were a predictor of hospital mortality, we used multivariable logistic regression to estimate the adjusted odds ratios (aORs) and 95% confidence intervals (CI) adjusting for age, gender, race/ethnicity, COPS2, LAPS2, admission care order, and direct intensive care unit (ICU) admission. All analyses used Python version 3.7.3 (Python Software Foundation, Beaverton, OR) and we considered *P* < .05 values statistically significant.

## 3. Results

### 3.1. Cohort characteristics

Among 364,506 hospital admissions occurring through the ED, we identified 159,004 (43.6%) with SI and 205,502 (56.4%) with S3 (Table 1). Among those with SI, 40.9% (n = 65,067) were males with a median age of 68 years (IQR 53–80). Patients with SI exhibited a median COPS2 of 27 (IQR 10–57), a LAPS2 of 60 (IQR 40–83), and unadjusted inpatient mortality was 1.2%. Patients with S3 were older and sicker with a median age of 73 years (IQR 62–83), COPS2 of 55 (IQR 26–90), and LAPS2 of 92 (IQR 67–120); hospital mortality was 8.4%. In the combined SI and S3 cohort, the most common infection source defined by primary and secondary admission diagnosis codes was of mixed type (44.7%), followed by respiratory (21.8%), bone, skin, or soft tissue (10.2%), and genitourinary (10.0%).

### 3.2. Antibiotic treatments

A total of 8792 unique combinations of antibiotics were administered across individual hospital encounters. In both SI and S3, the median duration of antibiotics was 3 days (IQR 2–5) with each patient receiving a median of 2 (IQR 1–3) Spectrum Score antibacterial groups. Overall, the most commonly administered antibiotic regimens were: 3^rd^ generation cephalosporins (8.3%), antipseudomonal fluoroquinolones (7.4%), 3^rd^ generation cephalosporins with macrolides (5.4%), 1^st^ generation cephalosporins (4.8%), and piperacillin-tazobactams (3.7%; Table 2).

**Table 2:** Most Common Antibiotic Combinations During the Entire Hospital Encounter

Overall (n = 364,506)	(%)	Suspected Infection (n = 159,004)	(%)	Sepsis (n = 205,502)	(%)
3rd Generation Cephalosporin	8.3	antiPseudomonal Fluoroquinolone	8.4	3rd Generation Cephalosporin	8.6
antiPseudomonal Fluoroquinolone	7.4	3rd Generation Cephalosporin	7.8	antiPseudomonal Fluoroquinolone	6.6
3rd Generation Cephalosporin, Macrolide	5.4	3rd Generation Cephalosporin, Macrolide	5.9	3rd Generation Cephalosporin, Macrolide	5.0
1st Generation Cephalosporin	4.8	1st Generation Cephalosporin	5.7	1st Generation Cephalosporin	4.0
Piperacillin-Tazobactam	3.7	3rd Generation Cephalosporin, Tetracycline	3.7	Piperacillin-Tazobactam	3.8
3rd Generation Cephalosporin, Tetracycline	3.4	Piperacillin-Tazobactam	3.6	Vancomycin, Piperacillin-Tazobactam	3.8
Piperacillin-Tazobactam, Vancomycin	3.3	antiPseudomonal Fluoroquinolone, Metronidazole	3.5	3rd Generation Cephalosporin, Tetracycline	3.2
antiPseudomonal Fluoroquinolone, Metronidazole	2.6	Vancomycin	2.7	antiPseudomonal Fluoroquinolone, Piperacillin-Tazobactam, Vancomycin	2.6
Vancomycin	2.3	Tetracycline	2.7	Vancomycin	2.0
antiPseudomonal Fluoroquinolone, Piperacillin-Tazobactam, Vancomycin	2.1	Piperacillin-Tazobactam, Vancomycin	2.7	antiPseudomonal Fluoroquinolone, Metronidazole	1.9

### 3.3. Spectrum scores

**Figure 1.**
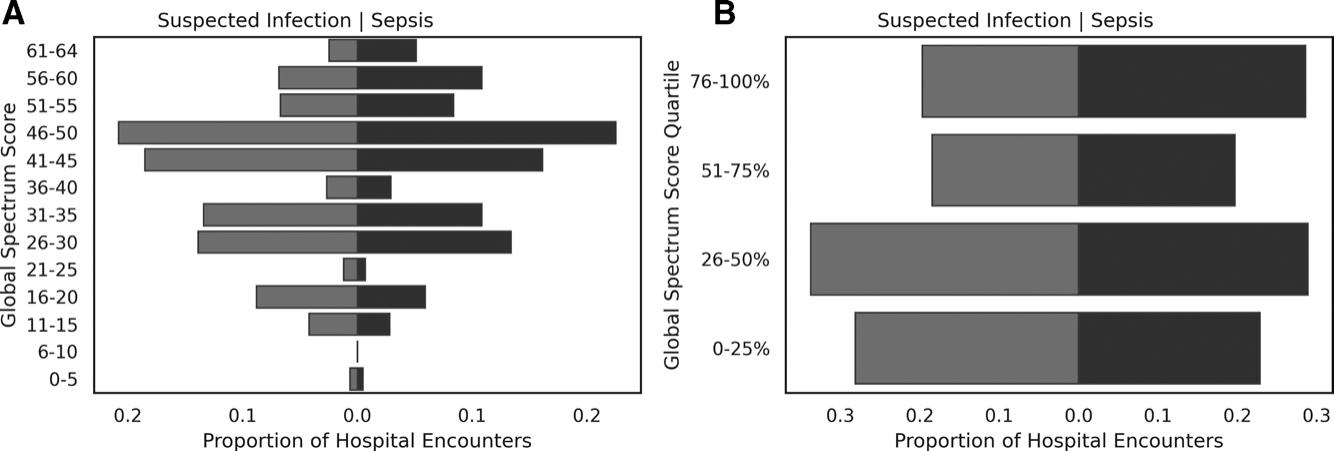
Histograms of global spectrum score and global spectrum score quartiles for hospital encounters in patients with suspected infection and sepsis. These figures show the proportion of hospitalized patients with suspected infection (left) and sepsis (right) grouped by Global Spectrum Score. Global Spectrum Scores were calculated from the combination of administered antibiotics within each hospital encounter (A). Global Spectrum Score quartiles were based on 25% cutoffs using Spectrum Scores from the entire cohort (B).

**Figure 2.**
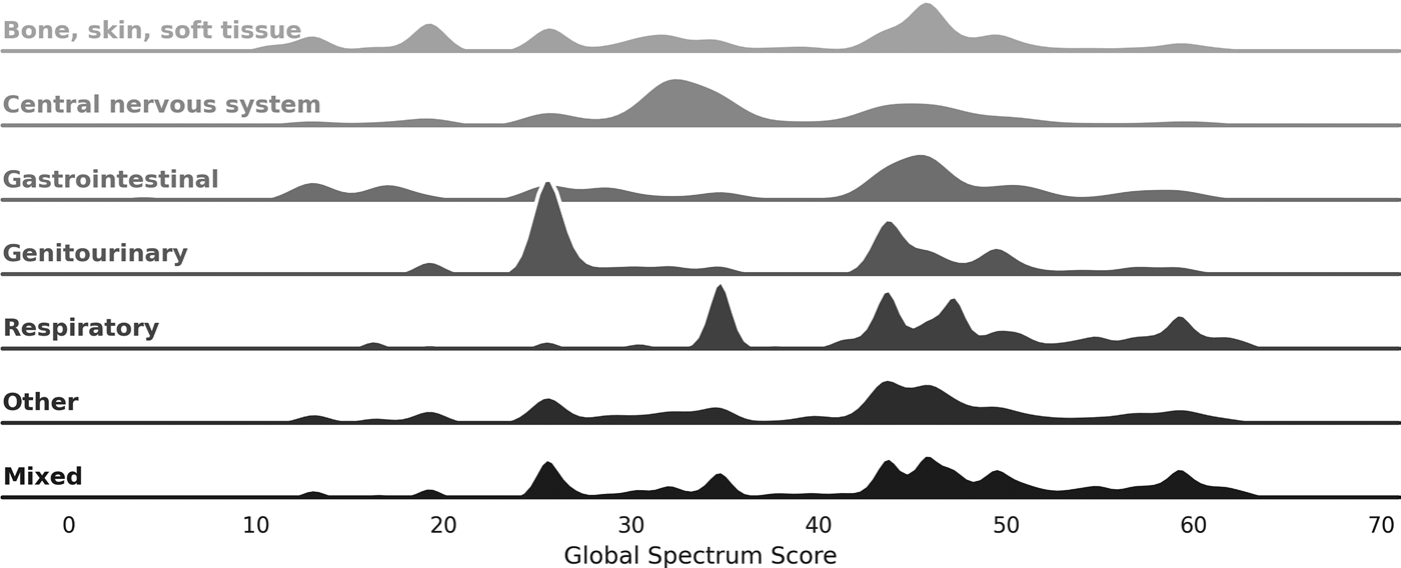
Ridge plots of global spectrum score by infection source. In these kernel density estimations, each horizontal plot represents a different infection source based on primary and secondary diagnosis codes (Table 1, Supplemental Digital Content, http://links.lww.com/MD/H100). The shape of each plot depicts the variability in antimicrobial breadth of exposure by global Spectrum Scores such as a tall peak at 25.5 for genitourinary infection sources or twin peaks at 43.8 and 47.3 for respiratory infection sources. While the genitourinary peak is driven by third generation cephalosporin usage and the respiratory peaks are driven by third generation cephalosporin with macrolide or antipseudomonal fluoroquinolone exposure, respectively.

Although the median global Spectrum Scores in S3 (43.5, IQR 26.8–47.2) were numerically similar to those with SI (43.8, IQR 32.0–49.5; *P* < .001; Table 1), S3 patients exhibited higher median additive Spectrum Scores (S3 114.0, IQR 57.0–204.5 vs SI 87.5, IQR 45.0–144.8; *P* < .001) (Fig. 1A). Fig. 1B also demonstrates that S3 patients were more frequently exposed to broad-spectrum antibiotics (defined empirically as a global Spectrum Score > 75^th^ percentile; Table 2, Supplemental Digital Content, http://links.lww.com/MD/H100) than those with SI (28.6% vs 19.6%; *P* < .001). Overall, median global Spectrum Scores were highest for respiratory (45.8, IQR 34.8–52.5) and mixed (45.8, IQR 34.0–51.5) infection sources, followed by other (43.5, IQR 30.3–48.0), gastrointestinal (43.5, IQR 25.5–47.0), genitourinary (38.5, IQR 25.5–45.8), bone, skin, and soft tissue (38.0, IQR 25.5–45.8), and central nervous system (34.5, IQR 32.0–45.8) infection categories. However, antibiotic breadth exhibited significant variability across infection sources as evidenced by multimodal peaks by hospital encounter (Fig. 2).

### 3.4. Changes in spectrum scores during hospitalization

**Figure 3.**
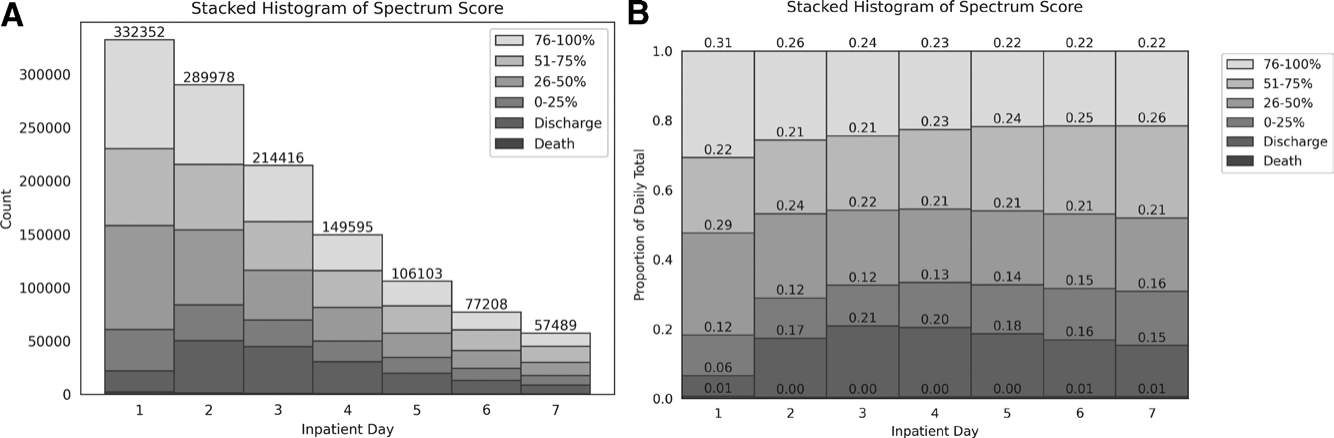
Stacked histograms of daily global spectrum score quartile in the first 7 days of hospitalization. In these stacked bar plots, each shaded bar (y-axis) represents the patients’ antibiotic exposure or outcome throughout the course of the hospitalization (x-axis). The height of each vertical shaded bar in Figure 3A represents the number of patients in each group and depicts a numeric reduction in antibiotic exposure as patients are discharged from the hospital or de-escalated off antibiotics. The height of each vertical-colored bar in Figure 3B represents the proportion of patients in each group and depicts a proportional reduction in antibiotic exposure from decreases in the broadest antibiotic exposure group (76–100%) as well as increases in the narrowest antibiotic exposure group (0–25%).

Figure 3 displays Spectrum Score value changes over the first 7 days of hospitalization by categories. The denominator for each column is the total number of patients in the hospital on that day and the numerator is the number of patients in each category. In the first 24 hours of hospitalization, nearly a third (34%) of patients were on broad-spectrum antibiotics which decreased to 22% by hospital day 7 regardless of SI or S3 (Fig. 3B). Overall, there was a trend towards decreasing breadth of antibiotic use; however, a substantial proportion of patients continued to exhibit significant antibiotic use at day 7, with only 16% of patient receiving no or narrow spectrum antibiotics (defined empirically as a global Spectrum Score ≤ 25^th^ percentile). Similar trends in Spectrum Scores were seen among SI or S3 cohorts (Figure 1, Supplemental Digital Content, http://links.lww.com/MD/H101).

### 3.5. Association between Spectrum Score and inpatient mortality

Higher antibiotic global Spectrum Scores were associated with an increased risk for hospital mortality (unadjusted OR per 10-point increase in Spectrum Score 1.59; 95% CI 1.57–1.61) (Table 4, Supplemental Digital Content, http://links.lww.com/MD/H100). This association remained even after adjusting for severity of illness metrics and patient demographics (1.34, 95% CI 1.32–1.36) as well as for source of infection (1.31, 95% CI 1.29–1.33).

## 4. Discussion

While there has been an intense focus on the early treatment of sepsis patients with antibiotics, corresponding tools to quantify and compare the breadth of these antibiotics has been lacking. In this study, we applied a previously developed metric of antibiotic breadth, the Spectrum Score, to a large, contemporary cohort of suspected infection and sepsis patients hospitalized through the ED. By using routinely available antibiotic administration data in the electronic health record, the Spectrum Score allowed us to compare the breadth of antibiotic exposure among individual patients, between suspected infection and sepsis populations, over the course of hospitalization, and across infection sources. In each of these comparisons, we found that there was considerable individual and temporal variability in antibiotic exposure which could be valuable to measure population-level antibiotic exposures and conduct research on the association between broad-spectrum antibiotic use and emerging resistance. The Spectrum Score could be used not only in the research setting but also in the clinical setting. Infectious disease specialists tasked with evaluating the appropriate use of broad-spectrum antibiotics could use the Spectrum Score as a rounding tool if it were embedded into their electronic medical record. Instead of searching for patients with sepsis diagnosis codes or rounding in the intensive care unit where patients may *appropriately* be on broad-spectrum antibiotics, the Spectrum Score could flag any patient in the hospital exposed to broad-spectrum antibiotics and prompt proactive consultation.

Existing literature suggests that as many as 50% of patients admitted to the hospital are exposed to antibiotics and nearly 13% of outpatient visits result in antibiotic prescriptions.^[12,13]^ In the outpatient setting, estimates suggest that over 30% of these outpatient prescriptions were inappropriate.^[13]^ In the inpatient setting, Magill and others recently suggested that antibiotic practices in community-acquired pneumonia or urinary tract infection diverged from guidelines in 55.9% of patients, raising the risk of substantial inappropriate use.^[14]^ At a patient level, even short courses of antibiotics can increase the risk of *C. difficile.*^[29]^ Patients exposed to antibiotics are also prone to microbiome perturbation or dysbiosis, potentially contributing to a higher risk of subsequent hospitalization for sepsis due to translocation or aspiration.^[30,31]^ The inappropriate use and overuse of antibiotics have also been identified as a tremendous global threat contributing to antimicrobial resistance and promotion of multi-drug resistant organisms.^[32]^

While limiting the breadth and duration of antibiotic exposure is a common principal of antibiotic stewardship programs, the lack of quantitative metrics contributes to challenges in comparing patients and populations treated with diverse and dynamic antibiotic regimens. Indeed, in our sample alone, we found that there were > 8000 unique combinations of antibiotics administered over the course of hospitalization. The Spectrum Score was designed to help standardize antibiotic exposures and could inform the impact of stewardship programs on antibiotic de-escalation and resistance patterns. Similarly, stewardship programs could incorporate scores to assess antimicrobial prescribing quality or appropriateness of antimicrobial use in healthcare systems.^[14,33]^ The scores may also have a role in improving outcomes when used in conjunction with procalcitonin-guided early discontinuation of antibiotics.^[34]^ Further, given the concerns about the intense focus on earlier antibiotics for sepsis, these tools can be used to assess whether sepsis programs drive the use of broader spectrum antibiotics unnecessarily.^[7]^

Unexpectedly, we found that patients with SI had similar median values for their global Spectrum Scores as those with S3. However, S3 patients did diverge in their additive Spectrum Scores which may have been primarily driven by longer overall duration of inpatient antibiotic use or some increase in the proportion of patients receiving broad-spectrum antibiotics. Because we remain uncertain about which of these factors—the single maximum breadth or the cumulative breadth of exposure—drives the adverse sequelae of antibiotic use, these tools offer a path towards defining which types of antibiotic de-escalation programs will offer the greatest benefits to patients. Several recent studies suggest that shorter courses of antibiotics are associated with improved patient outcomes, suggesting that reducing the duration of exposure alone may portend significant clinical benefits.^[35,36]^

While we report that global Spectrum Scores were associated inpatient mortality, even after adjusting for severity of illness and demographics, these results should be interpreted with caution due to residual confounding. Patients who are sicker or deteriorating are often exposed to broad-spectrum antibiotics, which is likely not accounted for in our adjusted analysis. However, these scores could be used to help understand thresholds for broadness of empiric coverage that maximize survival of sepsis and minimize adverse effects like *C. difficile.*^[15,29]^ More work needs to be done to understand the value or clinical use of Spectrum Scores.

Our study builds on previous studies in the field that evaluate antibiotic breadth by applying a validated method to a large cohort of patient with suspected infections and sepsis and is novel because we show variation using a numerical score of antibiotic exposure across suspected infection, sepsis, and infection source using the intrinsic antibacterial properties of each antibiotic agent. However, it is important to note that spectrum score tools are unlikely to be adequate when used as the only tool to evaluate antibiotic practice, because some agents like vancomycin have a spectrum score in the lowest quartile (e.g., <25^th^) yet may still have adverse effects similar to those from broad-spectrum agents.^[15]^ Thus, Spectrum Scores could be used within larger programs including those following Infectious Disease Society of American and Centers for Disease Control and Prevention guidelines.^[37,38]^

Our study also has several important limitations. While the Spectrum Score can account for various descriptions of antimicrobial coverage, it should be used in concert with other targeted approaches (e.g., prospective audit and feedback or antibiotic timeout^[39]^) to capture all aspects important to antibiotic use. Second, while we classified patients by infection source, patients were frequently diagnosed as having potential infections in multiple sites making it difficult to definitively establish a single source. Third, important factors such as recent antibiotic exposure, patient-specific organism sensitivities, and recent hospitalizations or use of nursing home were not captured in this study. Fourth, antibiotics can be used for other benefits outside of antimicrobial action (e.g., azithromycin for antiinflammatory effects in chronic obstructive pulmonary disease management^[40]^). We also did not account for the use of antibiotics beyond the hospitalization which are important considerations for population-level antibiotic management. Lastly, we did not evaluate the impact of antibiotic breadth on adverse patient outcomes or antimicrobial resistance patterns.

Our study has several strengths. The major strength is that it was done in a large, multi-center integrated healthcare system with a contemporary sample of patients using granular EHR data to quantitatively assess Spectrum Scores and outcomes. We were able to calculate longitudinal Spectrum Scores throughout hospitalization to determine cumulative and dynamic values. Another strength of this study was the capability to compare both SI and S3 across infection source to reveal underlying heterogeneity in antibiotic prescribing.

## 5. Conclusions

In a large and diverse sample of patients with sepsis and suspected infection, the use of Spectrum Scores quantifying antibiotic breadth revealed several sources of heterogeneity in antibiotic exposure among individual patients, between the suspected infection and sepsis populations, over the course of hospitalization, and across infection sources. This tool may offer an important quantitative metric for informing clinical prescribing patterns, guiding antibiotic stewardship programs, and evaluating the longer-term impacts of antibiotic practice.

## Acknowledgments

Statement about previous presentation of the data: Preliminary findings were presented online as abstracts at the American Thoracic Society International Conference 2021 and at the Critical Care Congress 2021.

## Author contributions

Dr. Smith had full access to all the data in the study and takes responsibility for the integrity of the data and the accuracy of the data analysis.

Conceptualization: Jones, Prescott, Liu.

Data curation: Smith, Manickam, Greene, Bhimarao, Jones.

Formal analysis: Smith, Jones, Myers, Prescott, Liu.

Funding acquisition: Prescott, Liu.

Investigation: Smith, Prescott, Liu.

Methodology: Smith, Pogue, Prescott, Liu.

Project administration: Barreda.

Resources: None reported.

Software: Smith, Manickam, Bhimarao.

Supervision: Prescott, Liu.

Validation: Smith.

Visualization: Smith.

Writing – original draft: Smith, Myers, Liu.

Writing – review & editing: Smith, Manickam, Barreda, Greene, Bhimarao, Pogue, Jones, Myers, Prescott, Liu.

## Supplementary Material


