# Supplementary material for: Quantifying the breadth of antibiotic exposure in sepsis and suspected infection using spectrum scores
Source: Medicine (Baltimore). 2022 Oct 14;101(41):e30245. doi: 10.1097/MD.0000000000030245 (PMC9575768; doi:10.1097/MD.0000000000030245)
Supplement: Supplementary file 1 [file medi-101-e30245-s001.pdf]

Quantifying the Breadth of Antibiotic Exposure in Sepsis and Suspected Infection Using Spectrum Scores

Supplementary Material Table of Contents

Supplementary Table 1. Workbook to Calculate Spectrum Score using Python

Supplementary Table 2. Descriptive Statistics of Spectrum Score by Quartile and Most Common Antibiotics

Supplementary Table 3. Grouping of ICD codes into Infection Source

Supplementary Table 4. Odds Ratios for Hospital Mortality Based on Maximum Spectrum Score During Encounter in Unadjusted and Adjusted Logistic Regression Models

Supplementary Figure 1. Stacked Histograms of Daily Global Spectrum Score Quartile in the First 7 Days of Hospitalization for Patients with Suspected Infection and Sepsis

References

This supplementary data has been provided by the authors to give readers additional information about their work.

Supplementary Table 1. Workbook to Calculate Spectrum Score using Python

https://colab.research.google.com/drive/1hseiQ81CgktL3SlwcUSJsHRHbk2Az4aL#scrollTo=Qc6jmhVfm8HY

Supplementary Table 2. Descriptive Statistics of Spectrum Score by Quartile and Most Common Antibiotics

Characteristic	Spectrum Score Quartiles			
	**0-25% (n=91,700)**	**26-50% (n=112,989)**	**51-75% (n=69,850)**	**76-100% (n=89,967)**
Unique patients	75,003	89,623	58,502	69,635
Global Spectrum Score, mean (SD)	21.5 (5.8)	38.8 (4.9)	46.4 (1.1)	55.5 (4.3)
Global Spectrum Score, median (IQR)	25.5 (18.0-25.5)	41.3 (34.8-43.8)	45.8 (45.8-47.3)	56.3 (50.8-59.3)
Global Spectrum Score, range	4.0-29.3	29.5-43.8	44.0-49.0	49.3-64.0
Most Common Antibiotic Combinations	3rd Generation Cephalosporin	Anti-Pseudomonal Fluoroquinolone	3rd Generation Cephalosporin, Tetracycline	Anti-Pseudomonal Fluoroquinolone, Piperacillin-Tazobactam, Vancomycin
	1st Generation Cephalosporin	3rd Generation Cephalosporin, Macrolide	Piperacillin-Tazobactam, Vancomycin	3rd Generation Cephalosporin, Anti-Pseudomonal Fluoroquinolone
	Vancomycin	Piperacillin-Tazobactam	Anti-Pseudomonal Fluoroquinolone, Metronidazole	Anti-Pseudomonal Fluoroquinolone, Vancomycin
	3rd Generation Cephalosporin, Metronidazole	Tetracycline	1st Generation Cephalosporin, Piperacillin-Tazobactam, Vancomycin	Anti-Pseudomonal Fluoroquinolone, Piperacillin-Tazobactam
	Macrolide	3rd Generation Cephalosporin, Vancomycin	3rd Generation Cephalosporin, Anti-Pseudomonal Fluoroquinolone, Vancomycin	3rd Generation Cephalosporin, Anti-Pseudomonal Fluoroquinolone, Macrolide

Supplementary Table 3. Grouping of ICD codes into Infection Source

**Infection Source**	**Healthcare Utilization Project (HCUP) clinical classification software (CCS) category numbers**
Bone, skin, or soft tissue	197, 198, 199, 201, 248
Central nervous system	76, 77, 78, 90
Gastrointestinal	6, 135, 148
Genitourinary	9, 156, 159
Respiratory	1, 92, 122, 123, 124, 125, 126, 129,134
Other	3, 4, 5, 7, 8, 97, 246, 247

Supplementary Table 4. Odds Ratios for Hospital Mortality Based on Maximum Spectrum Score During Encounter in Unadjusted and Adjusted Logistic Regression Models

Model	n	Odds Ratio for Hospital Mortality, per 10 points increase in Spectrum Score	95% CI
Unadjusted	364,506	1.59	1.57-1.61
+ Severity of illness and demographics[a]	363,116	1.34	1.32-1.36
+ Infection source[b]	255,817	1.31	1.29-1.33
Adjusted model in each cohort			
Suspected infection[a]	158,304	1.71	1.63-1.78
+ Infection source[b]	104,664	1.66	1.58-1.75
Sepsis[a]	204,812	1.29	1.27-1.31
+ Infection source[b]	151,153	1.26	1.24-1.28

[a]Adjusted logistic regression model includes age, gender, race, COPS2, and admission care order.

[b]Adjusted logistic regression model includes age, gender, race, COPS2, LAPS2, ICU admission, admission care order, and infection source.

Abbreviations: CI, confidence interval; COPS2, Comorbidity Point Score, version 2 [1]; ICU, intensive care unit; LAPS2, Laboratory Acute Physiology Score, version 2 [1]; OR, odds ratio.

References

1. Escobar GJ, Gardner MN, Greene JD, et al. Risk-adjusting hospital mortality using a comprehensive electronic record in an integrated health care delivery system. Med Care 2013;51(5):446-53.
