# Supplementary figures and images for: Quantifying the breadth of antibiotic exposure in sepsis and suspected infection using spectrum scores
Source: Medicine (Baltimore). 2022 Oct 14;101(41):e30245. doi: 10.1097/MD.0000000000030245 (PMC9575768; doi:10.1097/MD.0000000000030245)

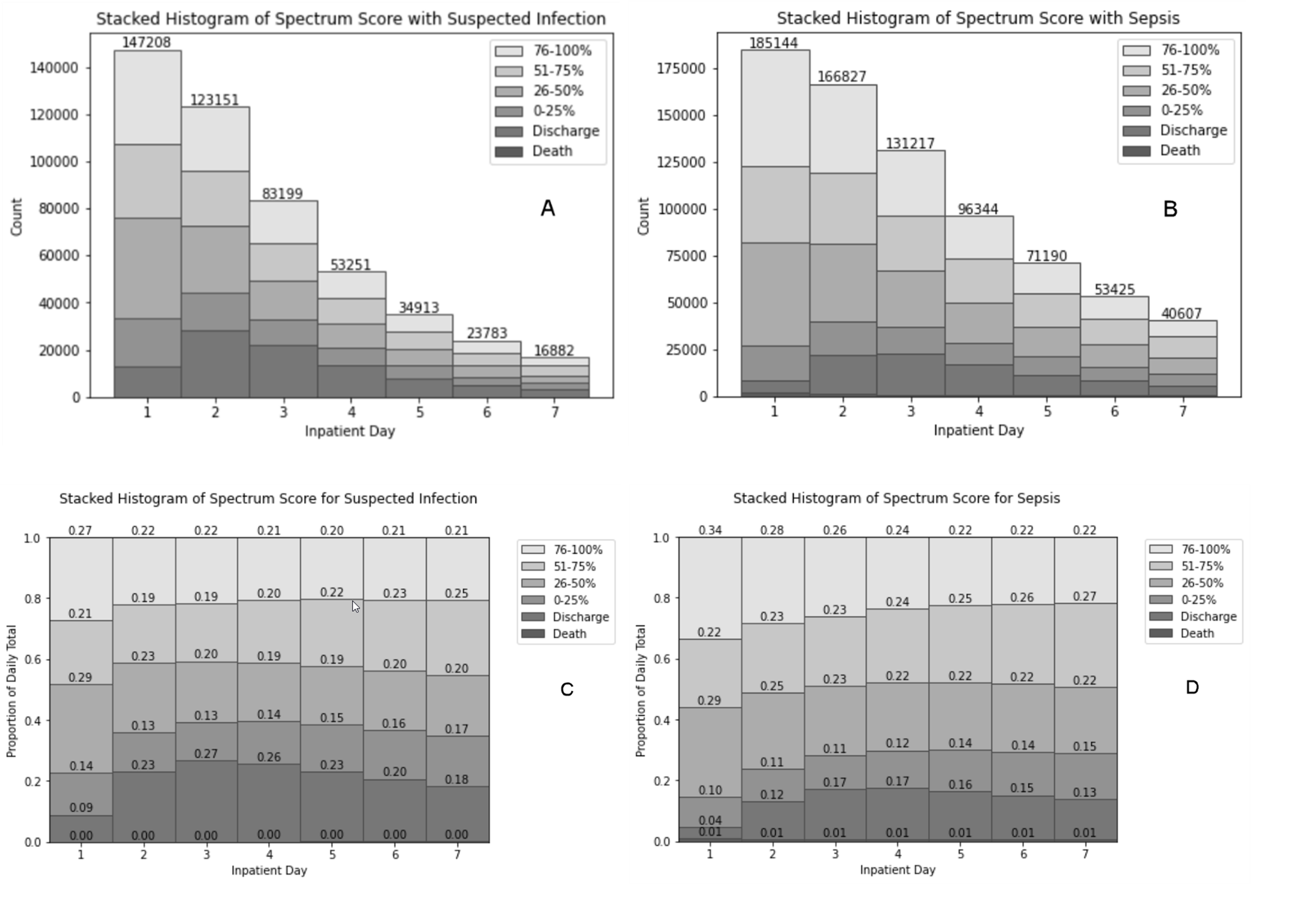

Supplement: Supplementary file 2 [file medi-101-e30245-s002.tif]
